# Impact of ice pack quantity and external temperature on chicken meat freshness during distribution in insulated boxes

**DOI:** 10.1007/s44463-025-00008-x

**Published:** 2026-02-11

**Authors:** Bu-Min Kim, Gyeong-seok Kang, Eun-Seon Lee, Jong-Hui Kim, Donghyun Shin, Mi-Hwa Oh

**Affiliations:** 1 Rural Development Administration, National Institute of Animal Science, Wanju, 55365 Korea; 2 Department of Agricultural Convergence Technology, Jeonbuk National University, Jeonju, 54896 Republic of Korea

**Keywords:** Chicken meat, Distribution, Ice pack, Storage temperature, Meat freshness

## Abstract

The proportion of livestock products purchased via online delivery has increased, emphasizing the need for standards to ensure the freshness and safety of perishable items like chicken. Microbial growth during chicken distribution is strongly influenced by external temperature, highlighting the importance of proper temperature control. This study evaluated the effects of external temperature and the number and spatial arrangement of ice packs on chicken quality in insulated boxes under simplified online delivery conditions. Samples were stored with ice packs at refrigerated (2 °C), ambient (25 °C), and high (40 °C) temperatures. At ambient and high temperatures with two ice packs, the total viable counts (TVC), volatile basic nitrogen (VBN), and thiobarbituric acid reactive substances (TBARS) significantly increased (*p* < 0.05), exceeding the freshness limits (6.7 log CFU/g for TVC, 20 mg% for VBN, and 0.20 mg MDA/kg for TBARS) by day 2. Spoilage also occurred when using four ice packs at ambient temperature, indicating their limited effectiveness in maintaining microbiological quality. Microbial indicators responded more sensitively to external temperature than chemical indicators and were affected by the spatial positioning of ice packs, indicating that optimized ice pack placement can delay spoilage. These findings emphasize the need for enhanced cold chain management strategies, including refrigerated vehicles and optimized thermal packaging. The absence of clear regulatory standards for temperature control during online meat distribution highlights the urgent need for institutional reforms to ensure the microbial safety and freshness of poultry products.

**Supplementary Information:**

The online version contains supplementary material available at 10.1007/s44463-025-00008-x.

## Introduction

Global meat consumption has steadily increased in recent decades. According to the OECD (2025), the average annual per capita meat consumption was approximately 40 kg worldwide in 2023, with significantly higher values observed in developed countries: 80.9 kg in the United States, 80.3 kg in Australia, and 53.7 kg in the European Union. Chicken, in particular, has become a major source of animal protein globally due to its cost-effectiveness (Sporchia et al., 2023).

Important factors to consider when purchasing chicken include freshness (32.7%), price (21.5%), country of origin (16.9%), and safety (14.5%) (KREI, 2024). Consumers primarily purchase chicken from large discount stores, corporate supermarkets, and general supermarkets. However, the proportion of online purchases has increased in recent years (Bae et al., 2020; KREI, 2024).

Since there is a delay between purchasing fresh meat online and its arrival to the consumer, maintaining proper storage conditions (e.g., temperature) during delivery is essential for food safety. Poor storage conditions can cause chicken spoilage due to the proliferation of various microorganisms (Rukchon et al., 2014), such as *Pseudomonas* spp. and *Shewanella putrefaciens* (Russell et al., 1995). Spoilage due to microorganisms causes an economic burden on chicken distributors and sellers and poses a health threat to consumers (Raeisi et al., 2016; Thompson et al., 2020).

Numerous studies have assessed the quality and freshness of chicken meat by analyzing volatile basic nitrogen (VBN) and thiobarbituric acid reactive substances (TBARS) under various controlled storage conditions (In’t Veld, 1996; Kim et al., 2021; Mikš-Krajnik et al., 2016; Yimenu et al., 2019). However, most previous studies have been conducted using static temperature settings, which do not adequately reflect the temperature variations that occur during actual distribution and transport. To address this limitation, further studies should assess chicken meat quality under dynamic external temperature conditions during distribution, including various cooling conditions.

Although the proportion of livestock products purchased through online delivery is increasing, consistent international standards and regulatory frameworks for cold-chain management remain a critical issue. Chicken, a representative white meat, is relatively more vulnerable to microbial growth and spoilage than red meat, and storage temperature and packaging conditions play a key role in maintaining chicken freshness (Nychas et al., 2008; Säde et al., 2013). According to the United State Department of Agriculture (USDA, 2024), fresh poultry must be stored and transported at a temperature no higher than 40℉ (4.4℃). Similarly, the European Union’s Regulation (EC) No 853/2004 (European Commission, 2004) requires that poultry meat must be kept at temperatures not exceeding 4℃ throughout storage and distribution. However, neither the United States nor the European Union has legally binding regulations specifically addressing temperature control during online delivery to consumers. Therefore, specific standards for delivery methods, including distribution temperature, are required to ensure chicken freshness and safety in both offline and online channels.

This study aimed to evaluate the effects of different external temperatures and ice pack quantity on the microbiological and physicochemical quality of chicken meat under simulated distribution conditions. The quality characteristics of chicken meat were evaluated under different storage temperatures by analyzing microbial counts, protein degradation (VBN), and lipid oxidation (TBARS) over time. By simulating varied distribution conditions, this study underscores the importance of adapting existing storage and transportation guidelines to reflect the evolving landscape of online food distribution.

## Materials and methods

### Experimental materials

**Fig. 1:**
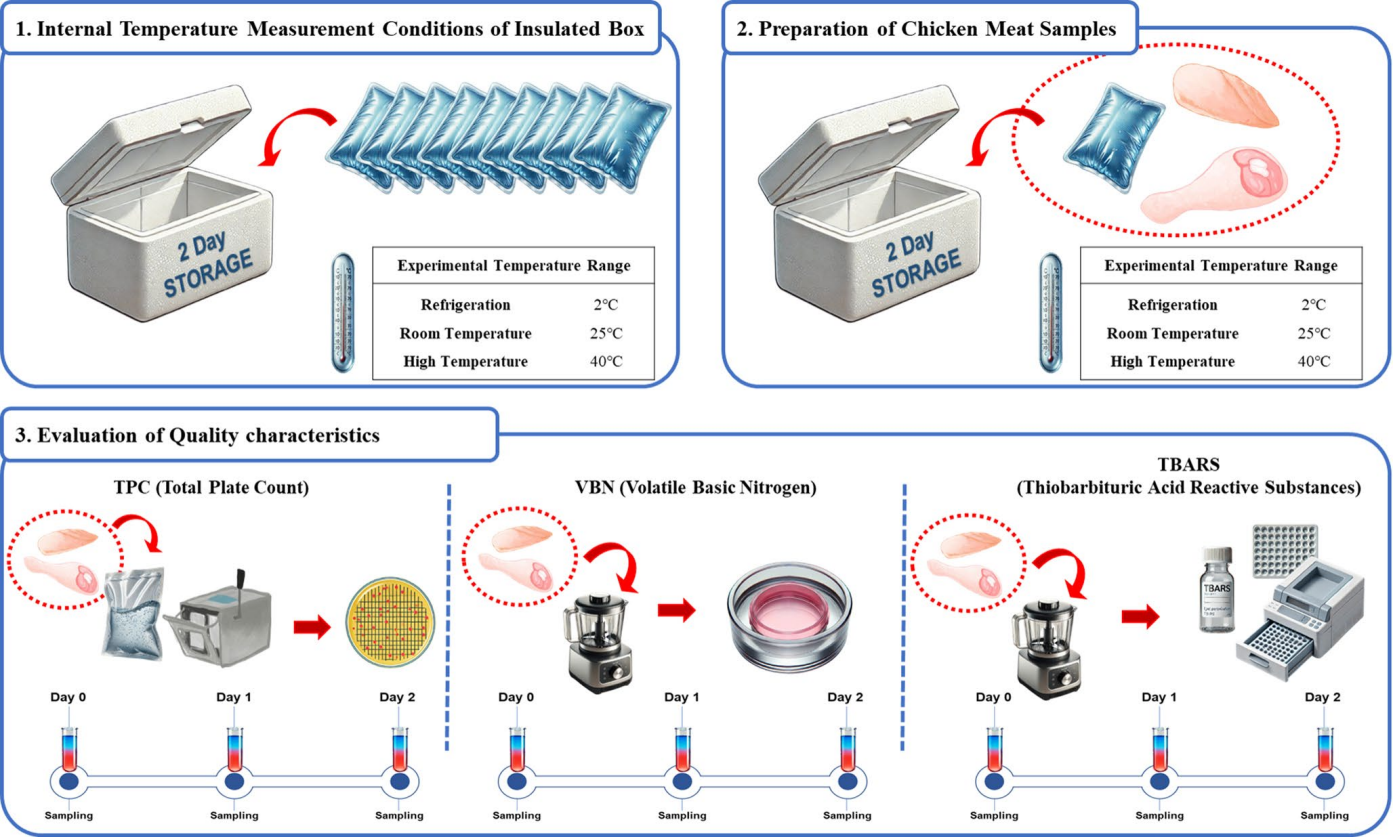
Flow diagram showing the experimental paradigm of the microbiological and physicochemical analyses

The chicken used in this study was purchased from the Harim Iksan Plant in Korea within 24 h of slaughter (951–1050 g, No. 14 according to the Korean Broiler Association standard). After skin removal, the chicken breasts and legs were aerobically packaged, and sampling was performed in three replicates. Two pieces of chicken breast and two pieces of chicken leg were considered as one sample for each individual, and the average weights of the samples used in the experiment were 275 g for chicken breast and 405 g for chicken leg. An insulated container (23 L styrofoam box measuring 50 × 35 × 20 cm) and 300 g ice packs (15 × 20 cm) were used to simulate typical packaging conditions for online chicken meat delivery. The microbial and physicochemical quality characteristics of the chicken were then analyzed (Fig. 1).

## Temperature monitoring inside an insulated box

A real-time temperature recording device (Thermo Recorder, TR-71nw, T&D Corporation, Japan) and ice packs were placed in each box under refrigerated (2℃), ambient temperature (25℃), and high-temperature (40℃) conditions. The boxes were then sealed with packing tape. The insulated box conditions were set according to the number and location of the ice packs, as shown in Tables 1 and 2, and the internal temperature changes were observed under each condition. Under refrigerated and high-temperature conditions, 0–4 ice packs were placed; for ambient temperature conditions, 0–9 ice packs were placed according to the experimental conditions. The temperature-recording device was placed on the wall 5 cm below the lid of the insulated box, and the internal temperature was measured every 15 min for 48 h. As a control, temperature was also monitored inside a refrigerator without the use of an insulated box.

**Table 1:** Ice pack treatment conditions in an insulated box under refrigerated and high-temperature environments

Sample name	Control	T0	2-T	3-T	4-T
No. of ice packs	0	0	2	3	4

Control, the temperature inside a refrigerator without the insulated box; all boxes (Control, T0, 2-T–4-T) were used to monitor internal temperature changes according to the number of ice packs; no chicken meat was included in this test

**Table 2:** Ice pack treatment conditions in an insulated box under ambient temperature

Sample name	Control	T0	2-T	3-T	4-T	5-T	6-T	7-T	8-T	9-T
No. of ice packs	0	0	2	3	4	5	6	7	8	9

Control, the temperature inside a refrigerator without the insulated box; all boxes (Control, T0, 2-T–9-T) were used only to monitor internal temperature changes according to the number of ice packs; no chicken meat was included in this test

## Insulated box conditions in microbiological and physicochemical experiments

Based on the internal temperature profiles of the insulated boxes under different external temperatures, appropriate ice pack conditions (as shown in Tables 3 and 4) were established to simulate storage environments for subsequent quality assessments of the chicken meat. The insulated boxes were placed under refrigerated and high-temperature conditions with two ice packs and under ambient conditions with two, three, or four ice packs. The positions of the ice packs were categorized as follows: above the chicken samples, below them, or evenly distributed above and below. When the number of ice packs was odd, one 300 g pack was replaced by two 150 g packs, allowing for an even distribution at the top and bottom. For comparison, chicken samples in the control group were stored directly in a refrigerator without placement inside an insulated box.

**Table 3:** Experimental setup of ice pack number and placement in insulated boxes containing chicken samples under refrigerated and high-temperature conditions

Sample name		No. of ice packs	Ice pack placement
Control		0	-
T0		0	-
2 T group	2-T1	2	On top of the sample
	2-T2		Below the sample
	2-T3		Evenly distributed

Control, chicken samples were stored in a refrigerator, not inside the insulated box; chicken samples were included in this experiment

**Table 4:** Experimental setup of ice pack number and placement in insulated boxes containing chicken samples under ambient temperature conditions

Sample name		No. of ice packs	Ice pack placement
Control		0	-
T0		0	-
2 T group	2-T1	2	On top of the sample
	2-T2		Below the sample
	2-T3		Evenly distributed
3 T group	3-T1	3	On top of the sample
	3-T2		Below the sample
	3-T3		Evenly distributed
4 T group	4-T1	4	On top of the sample
	4-T2		Below the sample
	4-T3		Evenly distributed

Control, chicken samples were stored in a refrigerator, not inside the insulated box; chicken samples were included in this experiment

## Total viable count (TVC) measurement

A 10 g sample of chicken meat was obtained using a sterilized blade. The sample was then placed in a filter bag containing 90 mL of diluent and homogenized for 2 min. We performed serial dilutions, and 1 mL of each dilution was plated onto a Petri film (Aerobic Count Plate, 3 M, St. Paul, Minnesota, USA). The plates were incubated at 37 °C for 48 h, after which the number of colonies was counted and expressed as colony forming units (CFU)/g.

## VBN analysis

Protein spoilage in chicken meat was evaluated by measuring VBN content using a modified Conway micro-diffusion method (Pearson, 1968). A 10 g meat sample was homogenized with 50 mL of distilled water (DW) and filtered through a Whatman No. 1 filter paper. The filtrate (1 mL) was added to the outer chamber of the Conway Unit, and Conway’s borate buffer (1 mL) was placed in the inner chamber. Conway’s borate buffer was prepared by dissolving 5 g of boric acid (H₃BO₃) in 100 mL of ethanol and adding 5 mL of Conway’s reagent (0.066% w/v methyl red + 0.033% w/v bromocresol green in ethanol). The solution was brought to a final volume of 500 mL with DW and thoroughly mixed. The edges of the Conway dish and its lid were sealed with petroleum jelly. Saturated K₂CO₃ solution (1 mL) was added to the outer chamber, and the dish was carefully sealed and shaken gently. The Conway’s unit was incubated at 37 °C for 2 h. After the reaction, 0.01 N H₂SO₄ was added to the inner chamber until the color of the solution changed to light pink. The amount of added 0.01 N H₂SO₄ was used to calculate the VBN value using the following equation:

1 $$\:\mathrm{VBN}\left(\mathrm{mg}/\mathrm{100g}\right)\:\mathrm{=}\:\left(\mathrm{a}-\mathrm{b}\right)\times \mathrm{F}\times \mathrm{D} \times \mathrm{0.14} \times \mathrm{100/S}$$

where S is the weight of the chicken sample (g), a is the volume of 0.01 N H₂SO₄ used for the sample titration (mL), b is the volume of 0.01 N H₂SO₄ used for the blank (mL), F is the standardization factor of 0.01 N H₂SO₄, and D is the dilution factor.

### Measurement of lipid oxidation (TBARS)

Lipid oxidation in chicken meat samples was measured using a modified version of the method described by Pikul et al. (1989). A 10 g sample of chicken meat was mixed with 35 mL of 4% perchloric acid and 1 mL of butylated hydroxytoluene. The mixture was homogenized for 1 min using a homogenizer and filtered through Whatman No. 1 filter paper. A 5 mL aliquot of the filtrate was mixed with 5 mL of 0.02 M thiobarbituric acid (TBA) and heated in a water bath at 80 °C for 60 min. After cooling for 10 min, the obtained sample solution was measured for absorbance (OD) at 532 nm, and the TBARS value was calculated using the following equation:

2 $$\:\text{TBARS value}\left(\text{mg malondialdehyde}/\mathrm{kg}\right)\mathrm{=} {\mathrm{OD}}_{\mathrm{532nm}}\times \mathrm{5.5}$$

### Statistical analysis

The experimental results were subjected to analysis of variance using R (Version 4.3.3), and comparisons between treatment groups were tested at a 95% significance level using Tukey’s honestly significant difference test (*p* < 0.05).

## Results and discussion

### Internal temperature variation under different insulated box conditions

The internal temperature of the insulated boxes was measured under different external temperatures for 48 h to evaluate the effects of different ice pack quantities. The results are summarized in Table 5. Under refrigerated conditions (2 °C), the internal temperature of the boxes reached 10 °C within 15 min after packaging, regardless of the number of ice packs, and remained below 2 °C after 48 h (Fig. S1, Table 5). These results indicate that boxes stored under refrigerated conditions met the temperature requirements for poultry storage (40℉ or 4 °C) in accordance with the international standards, as specified by USDA and EU regulations (European Commission, 2004; USDA, 2024). Under ambient temperature (25 °C), the minimum internal temperature of the insulated boxes decreased with increasing number of ice packs; however, none of the boxes reached temperatures below 5 °C during the 48 h storage period (Fig. S2, Table 5). Internal temperatures dropped below 10 °C only when four or more ice packs were used, and the duration of temperatures below 10 °C increased with the number of ice packs. Nevertheless, even when nine ice packs were used, the internal temperature did not remain below 10 °C for more than 24 h. Under high temperature (40 °C), the internal temperature of the insulated boxes did not reach 10 °C regardless of the number of ice packs used (Fig. S3, Table 5). Although the minimum internal temperature decreased as the number of ice packs increased, the lowest temperature recorded with four ice packs was 11.9 °C. After 48 h, the internal temperature of the box reached 40.7 °C.

**Table 5:** Internal temperature changes in insulated boxes under various conditions

	Sample name	T0	2-T	3-T	4-T	5-T	6-T	7-T	8-T	9-T
	No. of ice packs	0	2	3	4	5	6	7	8	9
Refrigerated condition (2℃)	Initial temperature (°C, immediately after packaging)	23.2	22.3	22.0	21.9					
	Minimum temperature (°C)	0.6	0.4	0	−1.7					
	Time to reach below 10 °C (min)	15	15	15	15					
	Duration below 10 °C (h)	48	48	48	48					
	Temperature after 48 h (°C)	2.0	2.0	1.2	1.2					
Ambient temperature (25℃)	Initial temperature (°C, immediately after packaging)	22.8	22.7	23.2	23.3	22.1	22.3	23.1	23.0	23.2
	Minimum temperature (°C)	21.8	13.0	10.2	9.2	7.3	7.0	6.0	5.6	5.6
	Time to reach below 10 °C (min)	-*	-*	-*	30	15	15	15	15	15
	Duration below 10 °C (h)	0	0	0	4.75	13	17	18.75	20.25	23
	Temperature after 48 h (°C)	24.5	23.9	24.7	23.7	22.5	22.1	20.7	20.8	21
High temperature (40℃)	Initial temperature (°C, immediately after packaging)	22.4	23.1	20.5	20.1					
	Minimum temperature (°C)	22.4	13.1	13.9	11.9					
	Time to reach below 10 °C (min)	-*	-*	-*	-*					
	Duration below 10 °C (h)	0	0	0	0					
	Temperature after 48 h (°C)	40	40	40.2	40.7					

Shaded areas represent conditions not tested in this study because the number of ice packs was limited to four under refrigerated and high-temperature conditions. The internal temperature remained below 10 °C in the refrigerated setting even when using fewer ice packs. Increasing the number of ice packs beyond four in high-temperature conditions did not achieve an internal temperature below 10 °C, so further testing was deemed impractical

* The internal temperature of the insulated box did not drop below 10 °C under this condition

T0, treatment with an insulated box without ice packs (negative control); 2-T–9-T, treatment with an insulated box containing 2–9 ice packs; all boxes (T0, 2-T–9-T) were to monitor internal temperature changes according to the number of ice packs; no chicken meat was included in this test

**Fig. 2:**
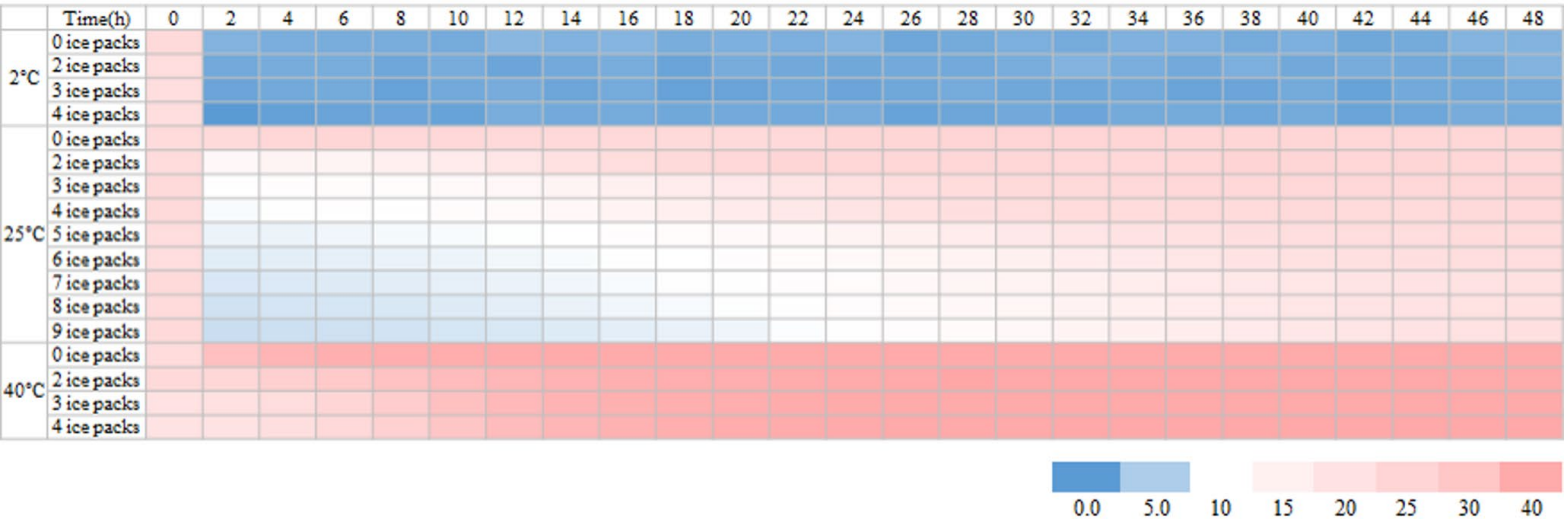
Temperature profiles in insulated boxes according to ice pack quantity and storage conditions. Temperature changes inside the insulated boxes were monitored over a 48 h period under three environmental conditions: refrigerated (2 °C), ambient (25 °C), and high temperatures (40 °C). Each condition was tested with various ice pack quantities (refrigerated: 0, 2, 3, and 4; ambient: 0–9; high temperature: 0, 2, 3, and 4). Color gradients represent internal temperature ranges: **blue** indicates temperatures ≤ 5 °C, **white** indicates 5–10 °C, and **red** indicates 10 °C

The internal temperature variations of the insulated boxes under different external environmental conditions (refrigerated, ambient, and high temperatures) and ice pack quantities are summarized in Fig. 2. Under refrigerated conditions, the internal temperature of the box was consistently below 10 °C, regardless of whether ice packs were used. In contrast, maintaining a refrigerated temperature inside a box at ambient or high temperatures is challenging even with ice packs. These results will help predict internal temperature changes in insulated boxes during online delivery. It can be inferred that when both refrigerated vehicles and ice packs are used, the internal temperature of the insulated box may drop below 10 °C within 15 min after packing and be maintained around 2 °C at the end of a 48 h storage period, which simulates the point of delivery (Fig. 2, Fig. S1). In contrast, when delivery is conducted without refrigerated transport, the ability of the insulated box to maintain a chilled temperature is limited. For instance, under ambient temperature, even though the internal temperature can initially drop below 10 °C when using four or more ice packs, it increases to 20.7–23.7 °C after 48 h of storage, which is unsuitable for the safe delivery of chicken meat (Fig. 2, Fig. S2). Furthermore, under high temperatures, such as in summer, the internal temperature of the box does not drop below 10 °C even with four ice packs, and it increases throughout the simulated delivery period (Fig. 2, Fig. S3).

Based on the internal temperature measurements of the insulated boxes, ice pack usage conditions were determined to evaluate the microbial and physicochemical quality of chicken meat. The results indicate that the number of ice packs did not significantly affect the internal temperature of the insulated box under each environmental condition (Fig. S1, Fig. S3). In the refrigerated condition (2℃), two ice packs were sufficient to maintain the internal temperature below 5℃. In contrast, under high-temperature conditions (40℃), adding more than four ice packs failed to reduce the internal temperature below 10℃. Therefore, for microbial and physicochemical quality assessments under these two temperature conditions, the number of ice packs was limited to two. In contrast, at ambient temperature, the initial internal temperature of the box decreased with increasing number of ice packs. Thus, two to four ice packs were used to assess the quality characteristics of chicken meat at ambient temperature. However, adding more than five ice packs (approximately 1.5 kg) could result in the total weight of the box exceeding that of the chicken meat itself, potentially causing inconvenience during transportation. Therefore, such excessive ice pack conditions were excluded from the experiment. The experimental packaging conditions, which were established by considering both the internal temperature control and the total weight of the insulated box, are listed in Table 5.

### Changes in microbial counts under different insulated box conditions

The changes in total microbial counts in chicken meat stored in the insulated boxes under various conditions are summarized in Table 6. Under refrigeration (2 °C), the TVC values of chicken breast in the refrigerated control group significantly increased during storage (*p* < 0.05), whereas the values for chicken legs slightly increased after day 1 but generally remained stable throughout the storage period. The TVC values for the chicken breast and leg samples stored in the insulated boxes without ice packs (T0) were similar to those in the control group. In the treatment group with two ice packs in the insulated box (2-T), the TVC of the chicken breast significantly increased over time, but at day 2 it was not significantly different from that of the control group for the same period. For chicken leg group treated with two ice packs (2-T), the microbial count remained relatively stable or slightly decreased during the first 2 d of storage. It was significantly lower than the control group over the same period. This phenomenon may be attributed to the combined effects of nutrient availability and the cooling environment provided by the ice packs, which influenced microbial growth and community formation (Ellis & Goodacre, 2001). In the refrigerated environment, the internal temperature of the insulated boxes with two ice packs was lower than that of the control group without ice packs (Fig. S1), which may explain the observed differences in microbial growth between the 2-T (chicken breast and legs) and control groups (Table 6). Differences in dominant microbial communities and nutrient composition between the chicken breast and leg samples, even under the same cooling conditions, may have influenced the characteristics of microbial growth. The protein and fat contents of the chicken breast samples was approximately 21.6% and 2.1%, respectively, whereas those of the chicken leg samples were approximately 18.0% and 7.4% (Bhawana et al., 2023). Chicken breast, given its high protein and low fat contents, is more conducive to the growth of proteolytic bacteria such as *Pseudomonas* spp. and *Brochothrix thermosphacta*, whereas chicken legs, given their high fat content, provide a more favorable environment for the growth of lipolytic bacteria such as *Acinetobacter* spp., *Psychrobacter* spp., and *Shewanella* spp. (Dourou et al., 2021). The chicken breast and leg samples in the 2-T group showed no significant differences (*p* < 0.05) or significantly lower microbial counts compared with those in the refrigerated control group. These results are consistent with both international guidelines, which generally regard meat as acceptable when TVC is below 5–7 log CFU/g (Carl, 1975; Heinz and Hautzinger, 2007), and Korean regulations, which set the limit at 6.7 log CFU/g (MFDS, 2024). Typically, poultry with an initial microbial count of 3–4 log CFU/g is considered acceptable, whereas counts exceeding 7–8 log CFU/g indicate the end of shelf life (Dawson et al., 1995; Raeisi et al., 2016; Senter et al., 2000). Kim et al. (2016a) found that the number of microorganisms increased from 4.84 to 7.96 log CFU/g when chicken was stored at 4 °C for 6 d, showing a similar trend to this study.

**Table 6:** Changes in the total viable count (TVC) chicken meat under different external temperature and ice pack treatments unit: CFU/g

			Chicken breast			Chicken leg		
			0 day	1 day	2 day	0 day	1 day	2 day
Refrigerated condition (2℃)	Control		3.94 ± 0.03^Aa^	4.15 ± 0.06^Ba^	5.76 ± 0.07^Cab^	4.18 ± 0.05^Aa^	4.34 ± 0.04^Ba^	4.19 ± 0.04^Aa^
	T0		3.94 ± 0.03^Aa^	5.05 ± 0.03^Bb^	5.84 ± 0.02^Cab^	4.18 ± 0.05^Aa^	4.42 ± 0.04^Ba^	4.24 ± 0.03^Aa^
	2-T group	2-T1	3.94 ± 0.03^Aa^	5.14 ± 0.02^Bb^	6.25 ± 0.48^Ca^	4.18 ± 0.05^Aa^	4.10 ± 0.03^Ab^	3.98 ± 0.06^Bb^
		2-T2	3.94 ± 0.03^Aa^	5.38 ± 0.05^Bc^	5.59 ± 0.27^Bb^	4.18 ± 0.05^Aa^	3.83 ± 0.08^Bc^	3.80 ± 0.06^Bc^
		2-T3	3.94 ± 0.03^Aa^	5.63 ± 0.06^Bd^	5.48 ± 0.06^Cb^	4.18 ± 0.05^Aa^	3.91 ± 0.06^Bc^	3.94 ± 0.03^Bb^
Ambient temperature (25℃)	Control		3.94 ± 0.03^Aa^	4.15 ± 0.06^Ba^	5.76 ± 0.07^Ca^	4.18 ± 0.05^Aa^	4.34 ± 0.04^Ba^	4.19 ± 0.04^Aa^
	T0		3.94 ± 0.03^Aa^	6.08 ± 0.06^Bb^	8.45 ± 0.09^Cb^	4.18 ± 0.06^Aa^	6.83 ± 0.01^Bb^	8.73 ± 0.06^Cbc^
	2-T group	2-T1	3.94 ± 0.03^Aa^	6.76 ± 0.03^Bb^	7.90 ± 0.04^Cbc^	4.18 ± 0.07^Aa^	6.24 ± 0.03^Bc^	8.86 ± 0.05^Cb^
		2-T2	3.94 ± 0.03^Aa^	6.51 ± 0.05^Bc^	7.83 ± 0.04^Cbcd^	4.18 ± 0.08^Aa^	5.77 ± 0.03^Bd^	8.47 ± 0.07^Ccd^
		2-T3	3.94 ± 0.03^Aa^	6.46 ± 0.06^Bc^	7.65 ± 0.05^Cd^	4.18 ± 0.09^Aa^	5.67 ± 0.03^Bd^	8.72 ± 0.11^Cbc^
	3-T group	3-T1	3.94 ± 0.03^Aa^	4.30 ± 0.08^Bad^	7.10 ± 0.07^Ce^	4.18 ± 0.10^Aa^	5.38 ± 0.04^Be^	8.12 ± 0.08^Ce^
		3-T2	3.94 ± 0.03^Aa^	4.23 ± 0.14^Bad^	7.03 ± 0.05^Ce^	4.18 ± 0.11^Aa^	5.00 ± 0.04^Bf^	8.05 ± 0.11^Cefg^
		3-T3	3.94 ± 0.03^Aa^	4.54 ± 0.05^Be^	7.84 ± 0.15^Cbcd^	4.18 ± 0.12^Aa^	6.34 ± 0.06^Bc^	8.10 ± 0.06^Cef^
	4-T group	4-T1	3.94 ± 0.03^Aa^	4.38 ± 0.10^Bde^	6.94 ± 0.08^Ce^	4.18 ± 0.13^Aa^	5.61 ± 0.16^Bd^	8.24 ± 0.36^Cde^
		4-T2	3.94 ± 0.03^Aa^	3.87 ± 0.16^Af^	7.98 ± 0.11^Bb^	4.18 ± 0.14^Aa^	4.96 ± 0.08^Bf^	7.77 ± 0.06^Cg^
		4-T3	3.94 ± 0.03^Aa^	4.30 ± 0.04^Bad^	7.74 ± 0.05^Ccd^	4.18 ± 0.15^Aa^	4.99 ± 0.02^Bf^	7.80 ± 0.03^Cfg^
High temperature (40℃)	Control		3.94 ± 0.03^Aa^	4.15 ± 0.06^Ba^	5.76 ± 0.07^Cb^	4.18 ± 0.05^Aa^	4.34 ± 0.04^Ba^	4.19 ± 0.04^Aa^
	T0		3.94 ± 0.04^Aa^	7.86 ± 0.03^Bb^	8.01 ± 0.03^Ca^	4.18 ± 0.06^Aa^	6.49 ± 0.02^Bb^	7.85 ± 0.06^Cb^
	2-T group	2-T1	3.94 ± 0.05^Aa^	7.92 ± 0.03^Bb^	7.94 ± 0.02^Ba^	4.18 ± 0.07^Aa^	6.86 ± 0.02^Bc^	7.96 ± 0.03^Cbc^
		2-T2	3.94 ± 0.06^Aa^	7.91 ± 0.01^Bb^	7.91 ± 0.03^Ba^	4.18 ± 0.08^Aa^	6.85 ± 0.02^Bc^	8.10 ± 0.08^Cc^
		2-T3	3.94 ± 0.07^Aa^	7.93 ± 0.02^Bb^	7.97 ± 0.06^Ba^	4.18 ± 0.09^Aa^	6.78 ± 0.02^Bd^	7.52 ± 0.10^Cd^

^A–C^ Means with different superscripts in the same row are significantly different (*p* < 0.05)

^a–g^ Means with different superscripts in the same column are significantly different (*p* < 0.05)

Note: Statistical comparisons of the ice pack treatments (a-g) were conducted separately within each temperature condition (2℃, 25℃, and 40℃), and no direct comparisons were made across different temperature groups

Control, chicken samples were stored in a refrigerator, not inside the insulated box (positive control); T0, treatment with an insulated box without ice packs (negative control); 2-T–4-T groups, treatment with 2–4 ice packs placed either above (T1), below (T2), or evenly distributed above and below (T3) the chicken sample in an insulated box

Under ambient temperature (25 °C), the TVC of chicken meat stored in insulated boxes rapidly increased over time (Table 6). In the T0 group (no ice packs), the microbial counts of both the chicken breast and leg samples significantly increased (*p* < 0.05), exceeding 6 log CFU/g within 24 h, unlike that in the refrigerated controls. In the chicken breast 2-T group, the microbial counts also exceeded 6 log CFU/g by day 1, although they remained within the microbiological limits for fresh meat (except for 2-T-1 sample). In contrast, the chicken breast 3-T and 4-T groups had counts ranging from 6.94 to 7.98 log CFU/g, exceeding the acceptable limit. In the chicken leg 2-T, 3-T, and 4-T groups, the microbial counts remained below 6.34 log CFU/g on day 1 but increased to 7.77–8.86 log CFU/g by day 2, failing to meet microbiological standards. Kim et al. (1990) measured the Q_10_ value of processed chicken products to predict their shelf life under various temperatures, and the storage period of processed chicken products stored at 25 °C was confirmed to be 4 d, which differs significantly from the results of this study. The difference in results may be attributed to the differences between raw and processed chicken. The minimum internal temperatures in the box placed at ambient temperature were 13.0 °C (2-T), 10.2 °C (3-T), and 9.2 °C (4-T), and the temperatures at 48 h were 23.9 °C (2-T), 24.7 °C (3-T), and 23.7 °C (4-T), respectively (Table 5 and Fig. S2). Although increasing the number of ice packs further decreased the minimum internal temperature (Table 5, Fig. S2), none of the insulated boxes exposed to ambient conditions maintained temperatures below 5 °C for 48 h. This may have contributed to the continued increase in microbial counts. If chilled chicken is transported at ambient temperature, even with ice packs, it may be challenging to ensure microbial quality and safety at the time of delivery (48 h mark).

Under high temperature (40 °C), the microbial counts in the T0 and 2-T groups significantly increased over time (*p* < 0.05), and most samples, except for the samples in the chicken leg T0 group, exceeded microbiological quality limits by day 1 (Table 6). Unlike the insulated boxes stored under refrigeration, the internal temperature of the boxes exposed to high temperatures never dropped below 10 °C, regardless of the number of ice packs used (Table 5, Fig. S3), and reached nearly 40 °C after 24 h of storage.

Although few studies have examined microbial growth in chicken stored at temperatures above 30 °C, Kim et al. (1990) reported that the microbial counts in processed chicken products remain below 10³ CFU/cm² by day 32 at 4 °C and 10 °C but increase to 10⁵ CFU/cm² at 25 °C by day 4 and 10⁶ CFU/cm² at 30 °C by day 2. This indicates that microbial spoilage progresses more rapidly at higher storage temperatures in processed chicken, which aligns with our findings of accelerated spoilage in raw chicken stored at 40 °C. In the present study, the chicken exposed to 40 °C may have experienced rapid quality degradation due to heat-induced protein denaturation. The continuous temperature increase inside the insulated box is also presumed to have promoted rapid microbial growth on the chicken surface. These results suggest that the microbiological quality of chicken is increasingly difficult to maintain when insulated boxes are continuously exposed to high ambient temperatures, such as during summer.

Furthermore, this study demonstrated that microbial growth dynamics in chicken meat were significantly influenced by the positioning of ice packs and the type of chicken parts. Although not consistently higher, TVC values in chicken legs tended to be greater than those in chicken breast under identical conditions, possibly due to their relatively higher myoglobin, fat, and moisture contents, which may create a more favorable environment for microbial proliferation (Bhawana et al., 2023; Noh et al., 2023). While may create a more favorable environment for microbial proliferation. While myoglobin itself does not directly stimulate microbial growth, its high iron content may promote oxidation and degradation processes, indirectly accelerating spoilage and microbial proliferation in leg meat compared to breast meat (Robach, 1961). In contrast to the double-sided ice-pack treatment (T3), the single-sided configurations (T1 and T2) generally exhibited a slower increase in TVC. This relative suppression of microbial growth may be associated with the partially open ice pack placement in T1 and T2, which likely influenced internal heat flow by allowing more effective dissipation of heat and facilitating localized air circulation. Although this interpretation remains speculative, it is supported by the comparative trends in microbial counts observed across the different packaging configurations.

### Changes in VBN levels under different insulated box conditions

In the postmortem period, nitrogenous compounds such as free amino acids, nucleic acid-related substances, amines, ammonia, and creatine are generated in meat over time due to endogenous proteolytic enzymes and microbial activity. Specifically, microbial enzymatic actions decompose proteins into amino acids, and further decarboxylation of these amino acids produces VBN compounds such as ammonia and trimethylamine (Field & Chang, 1969). As these compounds are byproducts of microbial protein degradation, VBN is widely used as a key indicator for evaluating meat freshness (Rukchon et al., 2014). While there is no internationally established standard for VBN levels in fresh meat in regions such as Europe or the United States, the Korean Food Code (MFDS, 2024) defines meat as spoiled if the VBN level exceeds 20 mg%.

The changes in VBN values of the control and treatment groups of chicken samples are shown in Table 7. Under refrigeration (2 °C), both chicken samples (breast and leg) maintained VBN values below 13 mg% over the 48 h period. The chicken breast control and T0 groups showed a significant increase in VBN values during the storage period, whereas the chicken breast 2-T group did not show any significant changes in VBN values over the 48 h storage period. In contrast, the chicken leg control group did not show significant differences in VBN values over the 2-day storage period, but the T0 and 2-T groups showed a significant increase in VBN values on day 2 compared with day 0. The difference in VBN increase trends between the chicken breast and leg samples may be attributed to compositional differences, as reported by Bhawana et al. (2023), who noted protein contents of 21.6% in chicken breast and 18.0% in chicken legs. This finding may explain the initially lower VBN levels in chicken legs than in the chicken breast. Thus, combining refrigerated transport systems with ice packs could help maintain chicken meat quality at levels similar to those achieved in conventional refrigeration, ensuring that VBN levels are below the spoilage threshold of 20 mg%.

**Table 7:** Changes in VBN content in chicken meat under different external temperature and ice pack treatments unit: mg%

			Chicken breast			Chicken leg		
			0 day	1 day	2 day	0 day	1 day	2 day
Refrigerated condition (2℃)	Control		10.71 ± 0.67^Aa^	11.94 ± 0.57^ABa^	12.46 ± 0.67^Ba^	8.25 ± 0.88^Aa^	8.43 ± 0.57^Aa^	8.89 ± 0.41^Aa^
	T0		10.71 ± 0.67^Aa^	12.05 ± 0.20^Ba^	11.94 ± 0.00^Bab^	8.25 ± 0.88^Aa^	9.36 ± 0.41^ABa^	9.95 ± 0.20^Bb^
	2-T group	2-T1	10.71 ± 0.67^Aa^	10.65 ± 0.20^Ab^	11.12 ± 0.20^Abc^	8.25 ± 0.88^Aa^	8.89 ± 0.41^ABa^	10.06 ± 0.20^Bb^
		2-T2	10.71 ± 0.67^Aa^	10.06 ± 0.20^Ab^	10.53 ± 0.00^Ac^	8.25 ± 0.88^Aa^	8.54 ± 0.20^ABa^	9.71 ± 0.20^Bb^
		2-T3	10.71 ± 0.67^Aa^	10.53 ± 0.00^Ab^	11.35 ± 0.20^Abc^	8.25 ± 0.88^Aa^	8.66 ± 0.41^ABa^	9.95 ± 0.20^Bb^
Ambient temperature (25℃)	Control		10.71 ± 0.67^Aa^	11.94 ± 0.57A^Bad^	12.46 ± 0.67^Ba^	7.90 ± 1.05^Aa^	8.60 ± 0.35^Aa^	8.60 ± 0.67^Aa^
	T0		10.71 ± 0.67^Aa^	16.94 ± 3.56^Ab^	28.44 ± 5.69^Bb^	7.90 ± 1.05^Aa^	14.74 ± 0.57^Bb^	42.83 ± 2.22^Cb^
	2-T group	2-T1	10.71 ± 0.67^Aa^	12.87 ± 0.20^Bacd^	30.19 ± 0.00^Cb^	7.90 ± 1.05^Aa^	12.52 ± 1.66^Bc^	26.91 ± 0.41^Cc^
		2-T2	10.71 ± 0.67^Aa^	15.91 ± 0.41^Bbc^	30.89 ± 3.97^Cb^	7.90 ± 1.05^Aa^	13.22 ± 0.88^Bbc^	22.82 ± 0.35^Cd^
		2-T3	10.71 ± 0.67^Aa^	14.74 ± 0.70^Babc^	24.92 ± 0.99^Cbc^	7.90 ± 1.05^Aa^	11.70 ± 0.41^Bc^	24.11 ± 0.41^Cd^
	3-T group	3-T1	10.71 ± 0.67^Aa^	11.58 ± 0.35^Aad^	11.58 ± 2.48^Aa^	7.90 ± 1.05^Aa^	9.36 ± 0.20^Aa^	16.27 ± 0.20^Be^
		3-T2	10.71 ± 0.67^Aa^	11.00 ± 0.73^Aad^	12.64 ± 0.99^Aa^	7.90 ± 1.05^Aa^	8.54 ± 0.20^Aa^	15.91 ± 0.41^Bef^
		3-T3	10.71 ± 0.67^Aa^	12.52 ± 0.88^Bacd^	15.10 ± 0.50^Cac^	7.90 ± 1.05^Aa^	9.24 ± 0.20^Aa^	21.65 ± 0.73^Bd^
	4-T group	4-T1	10.71 ± 0.67^Aa^	12.17 ± 0.20^Bacd^	12.99 ± 0.50^Ba^	7.90 ± 1.05^Aa^	9.13 ± 0.00^Aa^	13.46 ± 0.20^Bfg^
		4-T2	10.71 ± 0.67^Aa^	10.37 ± 0.32^Ad^	15.80 ± 0.50^Bac^	7.90 ± 1.05^Aa^	8.78 ± 0.61^Aa^	12.99 ± 0.61^Bg^
		4-T3	10.71 ± 0.67^Aa^	12.61 ± 0.18^Bacd^	12.99 ± 0.50^Ba^	7.90 ± 1.05^Aa^	8.66 ± 0.20^Aa^	17.32 ± 0.41^Be^
High temperature (40℃)	Control		10.71 ± 0.67^Aa^	11.94 ± 0.57A^Bad^	12.46 ± 0.67^Ba^	7.90 ± 1.05^Aa^	8.60 ± 0.35^Aa^	8.60 ± 0.67^Aa^
	T0		10.71 ± 0.67^Aa^	16.94 ± 3.56^Ab^	28.44 ± 5.69^Bb^	7.90 ± 1.05^Aa^	14.74 ± 0.57^Bb^	42.83 ± 2.22^Cb^
	2-T group	2-T1	10.71 ± 0.67^Aa^	27.15 ± 11.96^Ac^	292.54 ± 38.67^Bb^	7.90 ± 1.05^Aa^	35.46 ± 0.93^Bc^	342.86 ± 5.36^Cc^
		2-T2	10.71 ± 0.67^Aa^	24.57 ± 0.70^Bac^	342.86 ± 7.31^Cb^	7.90 ± 1.05^Aa^	27.73 ± 0.93^Bd^	348.71 ± 4.05^Cc^
		2-T3	10.71 ± 0.67^Aa^	26.80 ± 0.20^Ac^	292.54 ± 38.67^Bb^	7.90 ± 1.05^Aa^	28.32 ± 0.41^Bd^	340.52 ± 3.51^Cc^

^A–C^ Means with different superscripts in the same row are significantly different (*p* < 0.05)

^a–g^ Means with different superscripts in the same column are significantly different (*p* < 0.05)

Note: Statistical comparisons of the ice pack treatments (a-g) were conducted separately within each temperature condition (2℃, 25℃, and 40℃), and no direct comparisons were made across different temperature groups

Control, chicken samples were stored in a refrigerator, not inside the insulated box (positive control); T0, treatment with an insulated box without ice packs (negative control); 2-T–4-T groups, treatment with 2–4 ice packs placed either above (T1), below (T2), or evenly distributed above and below (T3) the chicken sample in an insulated box

Under ambient temperature (25 °C), the VBN levels in both chicken breast and leg samples significantly increased with storage time (Table 7). The VBN values in the chicken breast and leg T0 group rapidly increased, reaching 28.44 mg% and 42.83 mg%, respectively, on day 2. These results are consistent with previous findings where chicken breast stored at 20 °C exhibited VBN levels of 21.95 mg% (24 h) and 37.96 mg% (48 h) (Kim et al., 2022). In the chicken leg 2-T group, the VBN levels increased significantly over the storage period (*p* < 0.05), exceeding 22.82 mg%, showing a pattern similar to that observed in the breast samples. The VBN values in the chicken leg 3-T and 4-T groups also increased significantly with storage time. On day 1, the VBN levels in the 3-T and 4-T groups did not differ significantly from those in the chicken leg control group; however, by day 2, both groups showed significant differences compared with the control. These findings suggest that increasing the number of ice packs can effectively delay the rate of protein spoilage in chicken stored at ambient temperature.

At high temperatures (40 °C), the VBN levels in both chicken breast and leg samples also increased significantly over time (Table 7). On day 1, the VBN levels in the ice pack-treated groups were significantly lower than those in the T0 group, indicating some initial protective effect. However, by day 2, all treatments exceeded the threshold of 20 mg%, regardless of ice pack number or placement. This rapid increase in protein degradation is likely due to the proliferation of mesophilic bacteria such as *Bacillus* spp., *Clostridium* spp., and *Staphylococcus* spp. (Dave & Ghaly, 2011). These results suggest that the cooling capacity of ice packs is insufficient to preserve meat quality when stored at high temperatures and that insulated boxes alone do not ensure protein stability.

The VBN results showed that spoilage was affected by both the type of chicken parts and ice pack placement. As discussed in the microbial analysis, chicken legs showed a more rapid increase in spoilage indicators. This trend may be attributed to their distinct nutritional composition and the higher microbial activity associated with heme-derived compounds (Bhawana et al., 2023; Noh et al., 2023). Notably, the T3 treatment, where ice packs were placed both above and below the sample, consistently exhibited the highest VBN values across several treatment groups. This trend corresponds to the microbial results and supports the hypothesis that a semi-closed storage environment created by double-sided ice packs may have inadvertently facilitated microbial proliferation and proteolytic activity by limiting airflow. In contrast, the single-side icepack treatments (T1 or T2), generally showed more moderate increases in VBN. Based on the microbial and VBN data, it is possible that the partially open nature of these configurations allowed for better heat dissipation, thereby slowing the progression of spoilage. Interestingly, this pattern was more evident in chicken legs than in breast meat. In chicken breast, no clear differences in VBN levels was observed across different ice pack placements. As note earlier, this may be explained by the relatively lower susceptibility of breast to spoilage compared to chicken legs, likely due to its lower lipid content, reduced heme-associated reactivity, and comparatively lower microbial load.

In summary, the T1 and T2 treatments, with single-sided ice-packs, appeared to delay VBN accumulation. These findings, together with the microbiological results, suggest that ice pack configuration influences spoilage not only by controlling temperature but also by shaping the microenvironment that affects microbial growth and enzymatic protein degradation.

### Changes in TBARS under different insulated box conditions

Lipid oxidation is one of the major factors contributing to the deterioration of chicken meat quality. To evaluate the degree of lipid oxidation, the amount of malondialdehyde (MDA), a secondary byproduct of lipid peroxidation, was measured (Sujiwo et al., 2018). Although no international regulatory threshold for TBARS values in fresh meat has been established, Brewer et al. (1992) suggested that TBARS values should remain below 0.20 mg MDA/kg for meat to be considered fresh. Similarly, Kim et al. (2016b) proposed a threshold of 0.25 mg MDA/kg for processed meat.

The changes in TBARS values of the control and treatment groups of chicken samples are presented in Table 8. Under refrigerated storage (2 °C), the TBARS values of chicken samples increased significantly over time (*p* < 0.05), regardless of the number of ice packs. However, all samples exhibited values below 0.17 mg MDA/kg, indicating that lipid oxidation did not substantially contribute to quality deterioration under these conditions. Kim et al. (2021) reported that the TBARS values of chicken breast and legs stored at 4 °C increased from 0.13 to 0.14 mg MDA/kg and from 0.14 to 0.26 mg MDA/kg, respectively, over 3 d. In the present study, under refrigerated storage conditions, the TBARS values of chicken breast increased from 0.09 to 0.17 mg MDA/kg, and those of chicken legs increased from 0.08 to 0.16 mg MDA/kg, both showing similar trends to those reported by Kim et al. (2021). No significant differences in lipid oxidation were found between the control and treatment groups with or without ice packs (2-T vs. T0). These results suggest that maintaining insulated boxes in a consistently cold environment can help delay lipid oxidation to levels similar to direct refrigeration, even without the use of ice packs.

**Table 8:** Changes in TBARS content in chicken meat under different external temperature and ice pack treatments unit: Mg MDA/kg

			Chicken breast			Chicken leg		
			0 day	1 day	2 day	0 day	1 day	2 day
Refrigerated condition (2℃)	Control		0.09 ± 0.02^Aa^	0.09 ± 0.03^Aa^	0.16 ± 0.05^Ba^	0.08 ± 0.01^Aa^	0.09 ± 0.02^Aa^	0.12 ± 0.01^Ba^
	T0		0.09 ± 0.02^Aa^	0.12 ± 0.01^ABab^	0.13 ± 0.03^Ba^	0.08 ± 0.01^Aa^	0.09 ± 0.01^Aa^	0.16 ± 0.03^Ba^
	2-T group	2-T1	0.09 ± 0.02^Aa^	0.12 ± 0.01^Bab^	0.16 ± 0.02^Ba^	0.08 ± 0.01^Aa^	0.09 ± 0.01^Aa^	0.14 ± 0.04^Aa^
		2-T2	0.09 ± 0.02^Aa^	0.11 ± 0.01^ABab^	0.12 ± 0.02^Ba^	0.08 ± 0.01^Aa^	0.08 ± 0.01^Aa^	0.12 ± 0.03^Aa^
		2-T3	0.09 ± 0.02^Aa^	0.13 ± 0.01^ABb^	0.17 ± 0.05^Ba^	0.08 ± 0.01^Aa^	0.09 ± 0.02^Aa^	0.15 ± 0.04^Ba^
Ambient temperature (25℃)	Control		0.09 ± 0.02^Aa^	0.09 ± 0.03^Aa^	0.16 ± 0.05^Ba^	0.08 ± 0.01^Aa^	0.09 ± 0.02^Aa^	0.12 ± 0.01^Ba^
	T0		0.09 ± 0.02^Aa^	0.12 ± 0.01^ABab^	0.13 ± 0.03^Ba^	0.08 ± 0.01^Aa^	0.09 ± 0.01^Aa^	0.16 ± 0.03^Ba^
	2-T group	2-T1	0.08 ± 0.02^Aa^	0.17 ± 0.01^Bb^	0.23 ± 0.03^Cab^	0.09 ± 0.02^Aa^	0.21 ± 0.03^Bb^	0.27 ± 0.04^Bbc^
		2-T2	0.08 ± 0.02^Aa^	0.16 ± 0.01^Bb^	0.20 ± 0.04^Bab^	0.09 ± 0.02^Aa^	0.20 ± 0.01^Bb^	0.30 ± 0.02^Cbc^
		2-T3	0.08 ± 0.02^Aa^	0.16 ± 0.01^Bb^	0.25 ± 0.02^Ca^	0.09 ± 0.02^Aa^	0.20 ± 0.01^Bb^	0.30 ± 0.04^Cbc^
	3-T group	3-T1	0.08 ± 0.02^Aa^	0.09 ± 0.03^ABa^	0.14 ± 0.04^Bab^	0.09 ± 0.02^Aa^	0.12 ± 0.03^ABacd^	0.18 ± 0.06^Babc^
		3-T2	0.08 ± 0.02^Aa^	0.08 ± 0.04^Aa^	0.12 ± 0.05^Aab^	0.09 ± 0.02^Aa^	0.11 ± 0.00^Aad^	0.18 ± 0.04^Babc^
		3-T3	0.08 ± 0.02^Aa^	0.06 ± 0.03^Aa^	0.14 ± 0.04^Bab^	0.09 ± 0.02^Aa^	0.15 ± 0.03^ABbcd^	0.23 ± 0.08^Babc^
	4-T group	4-T1	0.08 ± 0.02^Aa^	0.09 ± 0.02^Aa^	0.10 ± 0.04^Ab^	0.09 ± 0.02^Aa^	0.11 ± 0.03^ABad^	0.16 ± 0.05^Bab^
		4-T2	0.08 ± 0.02^Aa^	0.07 ± 0.02^Aa^	0.15 ± 0.08^Aab^	0.09 ± 0.02^Aa^	0.10 ± 0.01^Aad^	0.20 ± 0.09^Babc^
		4-T3	0.08 ± 0.02^Aa^	0.08 ± 0.01^Aa^	0.13 ± 0.08^Aab^	0.09 ± 0.02^Aa^	0.13 ± 0.02^Aacd^	0.30 ± 0.07^Bc^
High temperature (40℃)	Control		0.09 ± 0.02^Aa^	0.09 ± 0.03^Aa^	0.16 ± 0.05^Ba^	0.08 ± 0.01^Aa^	0.09 ± 0.02^Aa^	0.12 ± 0.01^Ba^
	T0		0.09 ± 0.02^Aa^	0.12 ± 0.01^ABab^	0.13 ± 0.03^Ba^	0.08 ± 0.01^Aa^	0.09 ± 0.01^Aa^	0.16 ± 0.03^Ba^
	2-T group	2-T1	0.09 ± 0.02^Aa^	0.28 ± 0.13^Bb^	0.53 ± 0.03^Cb^	0.08 ± 0.01^Aa^	0.27 ± 0.04^Bc^	0.71 ± 0.05^Cc^
		2-T2	0.09 ± 0.02^Aa^	0.21 ± 0.04^Bab^	0.42 ± 0.05^Cc^	0.08 ± 0.01^Aa^	0.19 ± 0.01^Bd^	0.93 ± 0.02^Cb^
		2-T3	0.09 ± 0.02^Aa^	0.28 ± 0.05^Bb^	0.42 ± 0.02^Cc^	0.08 ± 0.01^Aa^	0.23 ± 0.01^Bcd^	0.71 ± 0.05^Cc^

^A–C^ Means with different superscripts in the same row are significantly different (*p* < 0.05)

^a–g^ Means with different superscripts in the same column are significantly different (*p* < 0.05)

Note: Statistical comparisons of the ice pack treatments (a-g) were conducted separately within each temperature condition (2℃, 25℃, and 40℃), and no direct comparisons were made across different temperature groups

Control, chicken samples were stored in a refrigerator, not inside the insulated box (positive control); T0, treatment with an insulated box without ice packs (negative control); 2-T–4-T groups, treatment with 2–4 ice packs placed either above (T1), below (T2), or evenly distributed above and below (T3) the chicken sample in an insulated box

Under ambient temperature (25 °C), the TBARS values of both chicken breast and leg samples gradually increased over 2 d, and the suitability of the meat based on lipid oxidation varied depending on the number of ice packs used (Table 8). The TBARS values in the chicken breast T0 and 2-T samples exceeded the 0.20 mg MDA/kg threshold by day 2. By contrast, the TBARS values in the 3-T and 4-T groups remained within 0.08–0.16 mg MDA/kg throughout the storage period, indicating lipid oxidation levels comparable to those in the refrigerated control group. Similar to the breast T0 and 2-T groups, the chicken leg T0 and 2-T groups exhibited TBARS values up to 0.30 mg MDA/kg on day 2, exceeding the freshness threshold. However, unlike in the breast samples, several chicken leg samples in the 3-T and 4-T groups (e.g., 3-T3, 4-T2, and 4-T3) also exceeded 0.20 mg MDA/kg. These differences are likely due to the varying fat contents between chicken breast and legs (Bhawana et al., 2023). Wazir et al. (2019) have similarly reported faster lipid oxidation and higher TBARS values in meat with high fat content. These findings confirmed that chicken legs, given their higher fat content, exhibit higher lipid oxidation than chicken breast.

Under high temperature (40 °C), the TBARS values in both chicken breast and legs increased rapidly over the 2 d storage period (Table 8). The TBARS values in all treatment groups exposed to high temperatures, except for chicken leg 2-T2 group, exceeded 0.20 mg MDA/kg by day 1, whereas that in the 2-T2 chicken leg group exceeded this value on day 2. The values increased to a range of 0.42–0.93 mg MDA/kg by day 2 in the ice pack treated group. These results indicate that under sustained high temperatures, ice packs and styrofoam insulation fail to control lipid oxidation. Increasing storage temperature accelerates MDA accumulation and lipid oxidation, leading to tissue damage and quality loss in meat (Wang et al., 2019; Wazir et al., 2019). In the present study, meat stored in insulated boxes continuously exposed to high temperatures underwent rapid lipid oxidation and significant quality deterioration. Therefore, even with ice packs, maintaining chicken meat quality during distribution is highly limited under inappropriate high-temperature environments.

Furthermore, under refrigeration and ambient conditions, no significant delay in lipid oxidation was observed in any of the chicken samples with respect to the ice pack positions. Under high-temperature conditions, most chicken samples exceeded the TBARS threshold from day 1, limiting any observable cooling effect based on the ice pack position.

### Spoilage indicators in response to cooling conditions

**Fig. 3:**
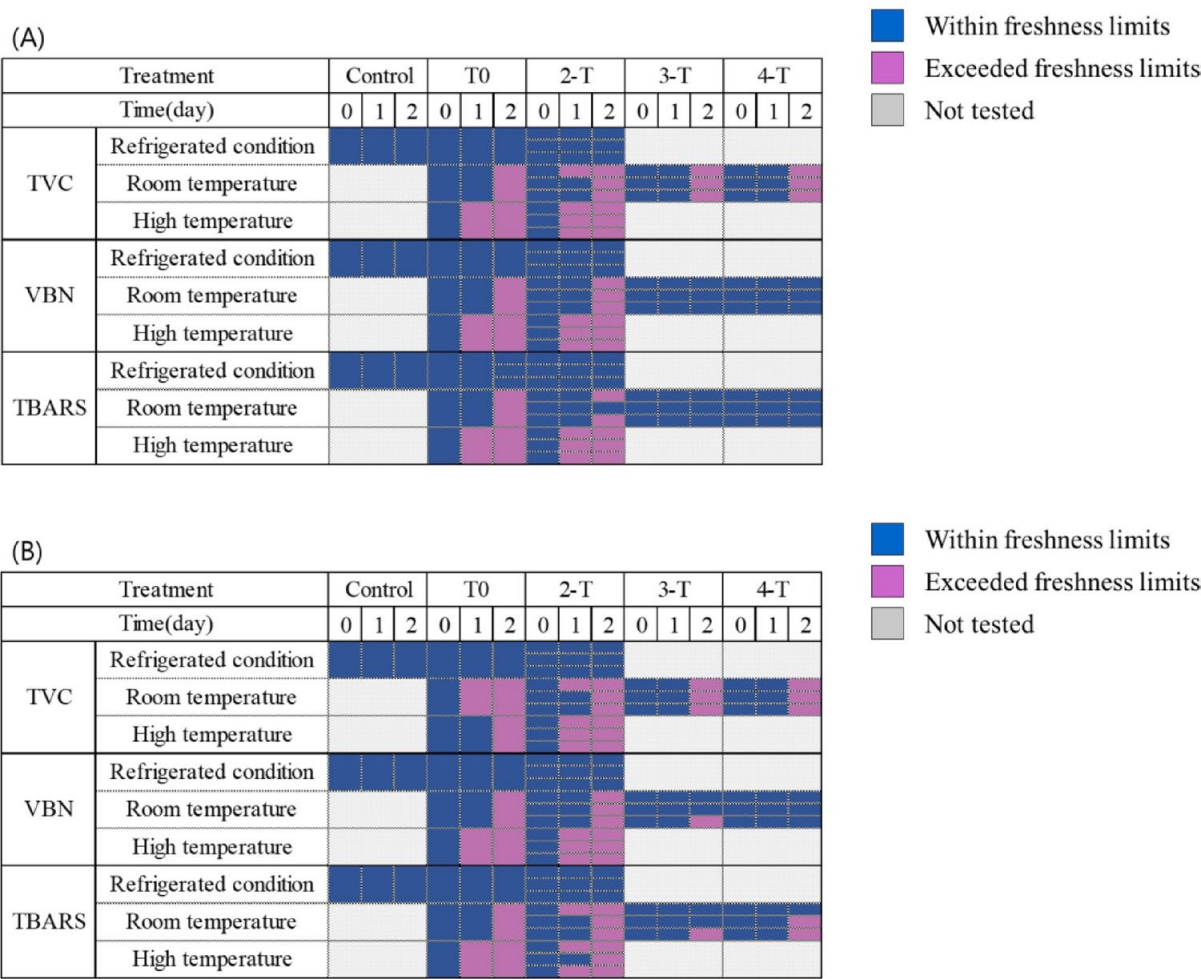
Effects of ice pack number and external temperature on chicken meat quality in insulated boxes. Chicken breast (**A**) and chicken legs (**B**). Control, chicken stored directly under refrigeration (2 °C) without an insulated box; T0, chicken stored in an insulated box without ice packs; 2-T, 3-T, and 4-T, chicken stored in insulated boxes containing 2, 3, or 4 ice packs, respectively. Quality indicators include total viable count (TVC), volatile basic nitrogen (VBN), and thiobarbituric acid reactive substances (TBARS). Color coding: blue, within freshness standards defined by national food regulations; pink, exceeding freshness limits; gray, not tested. Storage temperature conditions: refrigerated (2 °C), ambient temperature (25 °C), and high temperature (40 °C). This figure illustrates the combined effects of external temperature and ice pack number on chicken meat freshness over 0, 1, and 2 d of storage

The TVC, protein degradation (VBN), and lipid oxidation (TBARS) analyzed in this study are closely related to the quality of chicken meat. A comprehensive analysis of quality indicators depending on the ice pack numbers and external temperature (Fig. 3) revealed that, regardless of these variables, all samples exhibited earlier quality deterioration than chicken meat stored under direct refrigeration. Specifically, among the three key quality indicator, the TVC reflected changes in chicken meat quality more earlier than the other indicators in response to temperature fluctuations.

Although Fig. 3 does not present the effects of ice pack placement, previous observations described in the earlier section of this study suggest that TVCs were significantly affected by the position of ice packs, whereas VBNs (excluding the ambient stored chicken leg group) and TBARS did not show significant changes depending on ice pack location. This suggests that microbial growth is highly sensitive to the number of ice packs and their spatial distribution, which can cause localized thermal variation within an insulated container.

Under ambient temperature, when three or four ice packs were used, minimal deterioration was observed in the VBN and TBARS indicators over the first 2 d of storage, as shown in Fig. 3. In contrast, despite the chemical stability indicated by VBN and TBARS, the microbial count exceeded acceptable levels by day two, suggesting that microbial spoilage had already begun. This finding suggests that microbial growth is directly influenced by temperature fluctuations associated with the number of ice packs. Therefore, microbial indicators should be prioritized when assessing the quality of chicken meat.

Collectively, the results suggest that thermal distribution is based on the number and placement of ice packs and the external temperature. Among the three spoilage indicators examined, microbial growth showed the most rapid and sensitive response to temperature changes, underscoring its value as a key marker for quality assessment.

Effective temperature management to minimize microbial growth is essential to maintain quality during the delivery of chicken meat. However, the findings of this study indicate the limited effectiveness of managing microbial quality using ice packs alone under ambient temperature conditions. This highlights the need for more efficient refrigeration packaging technologies and a systematic transportation environment capable of maintaining a stable temperature during transit. Given that regulations lack clear temperature control standards, institutional improvements, including the requirement for refrigerated trucks during delivery, are urgently required to ensure the freshness of perishable meat products such as chicken. This study was limited by the use of a single type of ice pack and the application of simplified external temperature settings, which did not fully capture the range of temperature fluctuations that may occur during the distribution process. Further studies should evaluate cooling performance under variable external temperature conditions and with diverse cooling materials, considering day-night temperature changes and transient heat exposure during transport, to better simulate actual delivery environments.

## Conclusion

This study investigated the quality changes of chicken meat under simulated delivery conditions, focusing on the number and arrangement of ice packs. Chicken meat stored under refrigerated conditions in an insulated box maintained freshness within acceptable limits for two days, regardless of the number or position of the ice packs, similar to samples stored under direct refrigeration (control). In contrast, chicken samples stored with only two ice packs exhibited serious quality deterioration within two days under both ambient and high-temperature conditions. Even when four ice packs were placed in a 23 L insulated box at ambient temperature, the microbial counts exceeded the acceptable limits by day 2, indicating that ice packs alone cannot sufficiently suppress microbial growth under such conditions. Among the evaluated quality indicators, the microbial indicators were most sensitive to temperature changes, making them critical indicators of early quality deterioration. Furthermore, unlike chemical indicators such as VBN and TBARS, microbial indicators demonstrated their sensitivity to the spatial arrangement of ice packs. This suggests that optimizing ice pack placement can enhance thermal stability and delay spoilage in perishable foods. These findings underscore the critical importance of maintaining strict temperature controls to preserve the freshness and safety of chicken meat during delivery. Nevertheless, domestic regulations, including the Korean Food Code, lack clear guidelines specifically addressing temperature management in the context of online food delivery. Therefore, comprehensive improvements, including the mandatory use of refrigerated delivery vehicles and thermal packaging systems optimized for cooling performance, are required to ensure the quality of fresh meat products, particularly poultry, during online distribution. This study provides data-driven evidence to support such institutional and technological enhancements.

## Supplementary Information

Below is the link to the electronic supplementary material.

Supplementary Material 1

Supplementary Material 2

Supplementary Material 3
