# Supplementary figures and images for: Impact of ice pack quantity and external temperature on chicken meat freshness during distribution in insulated boxes
Source: Food Sci Anim Resour. 2026 Feb 11;46(1):30. doi: 10.1007/s44463-025-00008-x (PMC12995017; doi:10.1007/s44463-025-00008-x)

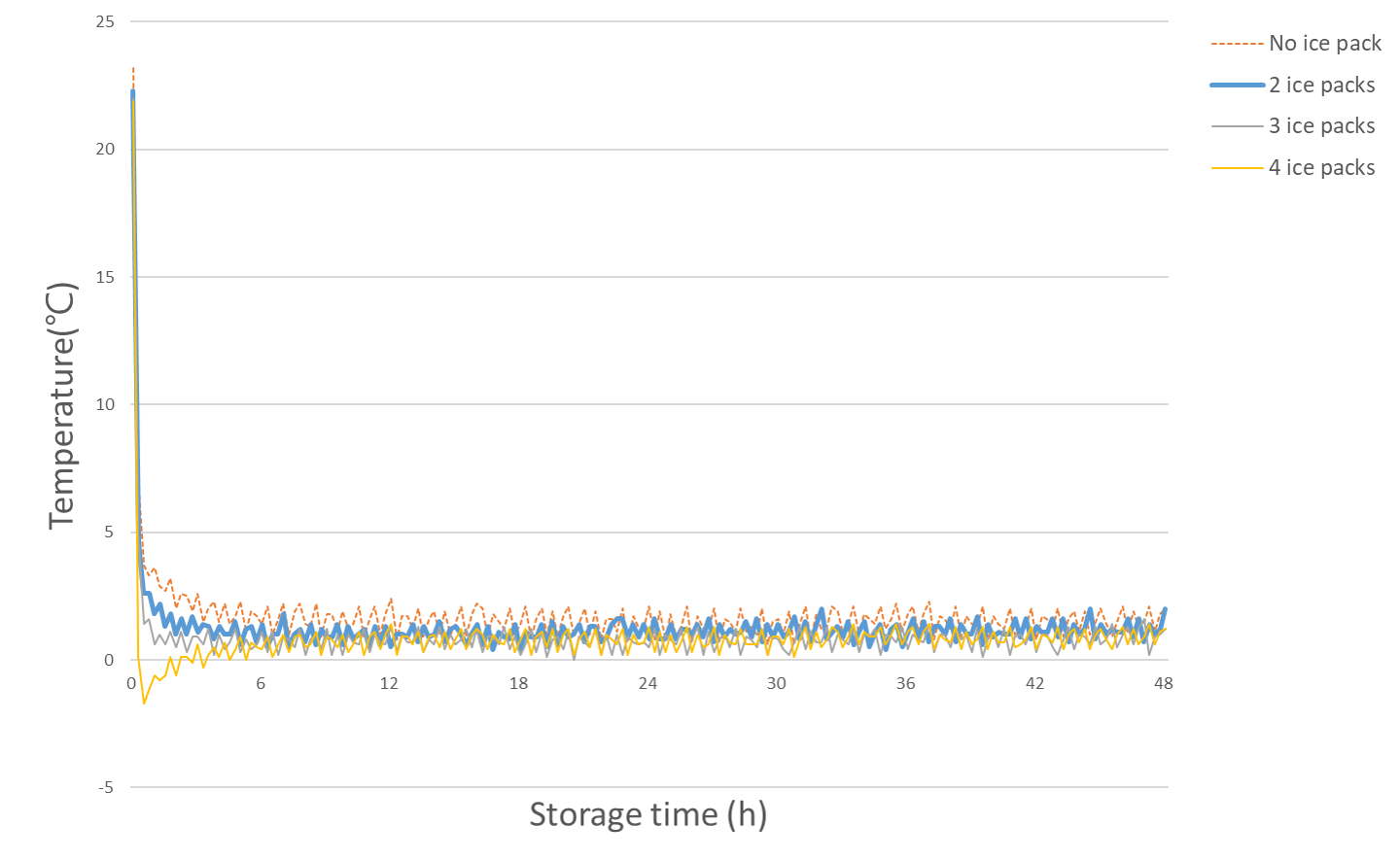

Supplement: Supplementary file 1 — Supplementary Material 1 [file 44463_2025_8_MOESM1_ESM.png]

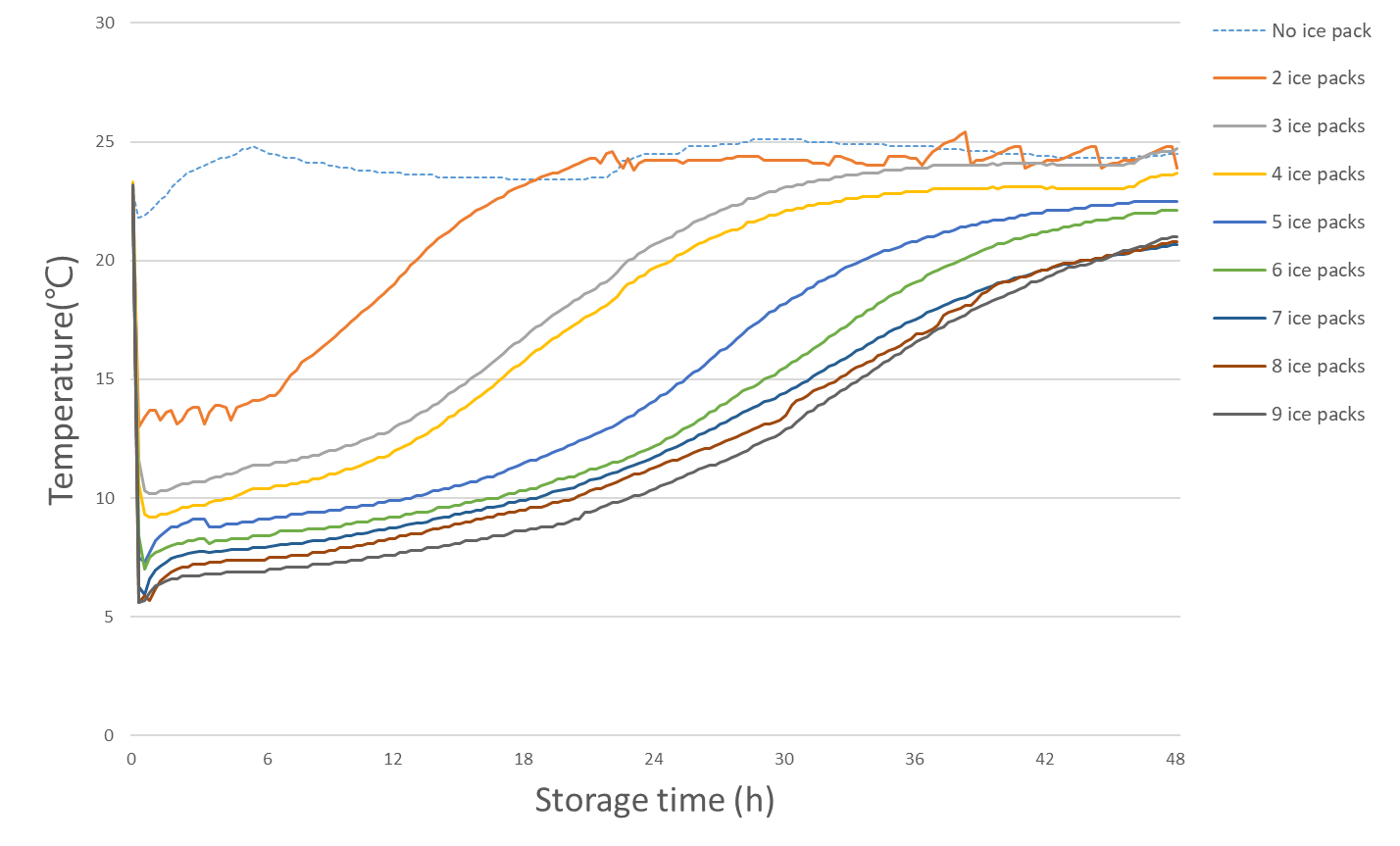

Supplement: Supplementary file 2 — Supplementary Material 2 [file 44463_2025_8_MOESM2_ESM.png]

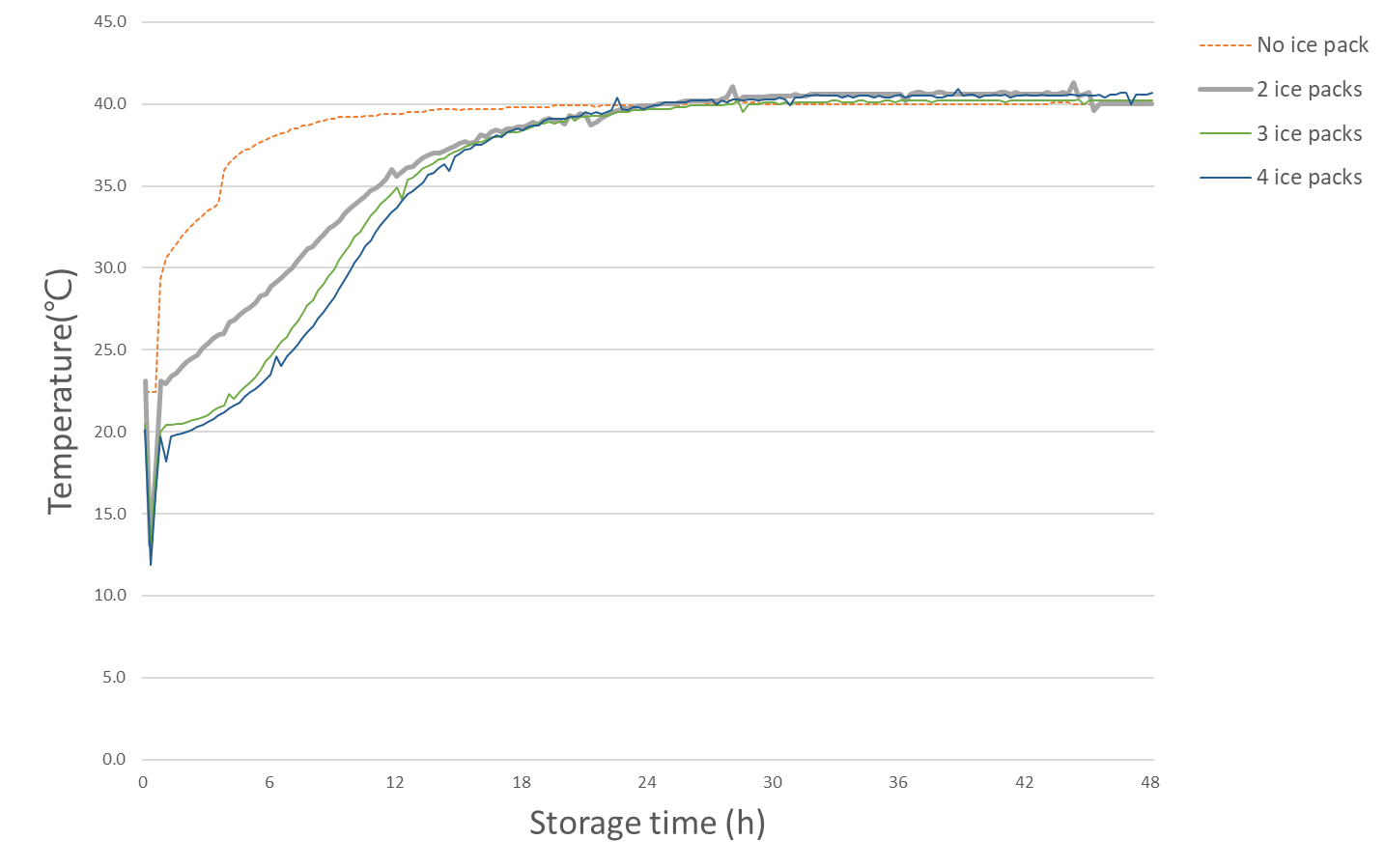

Supplement: Supplementary file 3 — Supplementary Material 3 [file 44463_2025_8_MOESM3_ESM.png]
